# Open reduction for developmental dysplasia of the hip: failures of screening or failures of treatment?

**DOI:** 10.1308/003588413X13511609957137

**Published:** 2013-03

**Authors:** AP Sanghrajka, CF Murnaghan, A Shekkeris, DM Eastwood

**Affiliations:** ^1^Norfolk and Norwich University Hospitals NHS Foundation Trust, UK; ^2^The Royal Hospital for Sick Children, UK; ^3^South-West Thames Radiology Specialist Training Rotation, UK; ^4^Great Ormond Street Hospital for Children NHS Foundation Trust, UK

**Keywords:** Congenital hip dislocation, Congenital hip dysplasia, Screening

## Abstract

**Introduction:**

The aim of this study was to define the clinical indications and demographic characteristics of patients undergoing open reduction for developmental dysplasia of the hip (DDH), and determine the proportion due to preventable failures of contemporary clinical screening and early management.

**Methods:**

Case notes were reviewed of consecutive primary open reductions performed for non-teratologic hip dislocation at the Great Ormond Street Hospital for Children over a five-year period. Forty-eight patients (64 hips) were suitable for inclusion. A telephone survey confirmed selective hip ultrasonography screening protocols were employed in all maternity hospitals in our referral base.

**Results:**

There were no cases of open reduction for unilateral DDH following Pavlik treatment commenced by six weeks of age, highlighting the importance of early detection and treatment. Eleven cases (23%) may have been avoided by appropriate implementation of existing selective ultrasonography screening protocols. Thirty-four cases (71%) presented after four months of age, suggesting open reduction is associated with late diagnosis rather than failure of primary management. None of these patients had neonatal hip ultrasonography and only 12% (4 patients) had a risk factor that should have triggered a scan.

**Conclusions:**

Compared with published results, the contemporary screening practices in our referral base are failing to eliminate late presenting DDH and the need for open surgical reduction. Changes in strategy and implementation are required to significantly improve screening efficacy.

Developmental dysplasia of the hip (DDH) is one of the most prevalent congenital abnormalities in newborn children.^1^ The neonatal hip has a tremendous potential for growth and remodelling. Reducing the femoral head into the acetabulum guides the growth of the acetabular depth and concavity, resulting in a normal hip. This forms the basis for conservative methods of management such as those of Pavlik or von Rosen, which have been shown to have excellent rates of success, with few complications.^2–4^

It is intuitive that early detection and treatment prevents the progression of anatomical abnormalities in a dysplastic hip, and also maximises the available growth and remodelling potential of the neonatal hip. Successful Pavlik harness treatment has been reported to restore hip development to normal, with considerably lower rates of osteonecrosis and secondary surgery than either open or closed reduction.^3–6^ The literature supports the importance of early detection with non-operative treatments such as the method of Pavlik, demonstrating less favourable outcomes with increasing age.^2,7^ Similarly, the outcomes of closed reduction are better when performed before the age of 12 months.^8^ Screening is important for the early detection of DDH, which facilitates closed methods of reduction, with the ultimate goal of avoiding open surgical reduction.

The implementation of a robust and well managed splintage regimen should result in success rates of about 90% in Graf type III and 60% in Graf type IV dysplasia/dislocation.^9,10^ The majority of the remainder will respond successfully to a closed reduction.^9–11^ Only a small proportion of these cases will therefore ultimately require an open reduction. Effective screening programmes have been reported to have significantly reduced the rates of all types of surgery for DDH (tenotomy, closed reduction, open reduction and osteotomies).^11–13^

Open reduction for hip dysplasia is indicated in those few cases where there has been either a failure of early management or failure of hip screening (resulting in late detection). No previous studies have investigated which is the most common indication. This is important as it has implications for understanding the efficacy of current screening practices. With effective screening, few hips would require open surgical reduction and all of those that do should be failures of early management.

The purpose of this study was to review our recent cases of open reduction for DDH with reference to the indication for surgery. We aimed to determine the relative proportions performed for failed early management and for late presentation, comparing the characteristics of patients in those two groups with respect to type of involvement (unilateral or bilateral), risk factors for DDH (female, first born, breech presentation, oligohydramnios, family history, history of intrauterine packaging disorder [eg congenital torticollis, metatarsus adductus])^14^ and history of neonatal ultrasonography examination. The information from our study could be used to inform criticisms of current screening practices.

## Methods

This study was performed at the orthopaedic department of the Great Ormond Street Hospital for Children, a tertiary-level paediatric hospital. The hospital provides neither maternity services nor routine screening of newborns, eliminating some sources of potential bias. Referrals are accepted from secondary centres; any child with a diagnosis of DDH will be seen and assessed for either non-surgical or surgical management.

This was a retrospective review of a consecutive case series of primary open reduction for DDH performed at our centre in the five-year period from 2004 to 2008. Cases were identified using the departmental database and theatre records.

A minimum dataset was completed for each patient (Table 1) from the case notes and telephone interviews with the parents. Patients with teratologic dislocations and those in whom we could not obtain the minimum required information were excluded from the study. The resulting data were analysed using a combination of qualitative and quantitative methods.

**Table 1:** Minimum dataset required per patient for inclusion in study

> Age at diagnosis of developmental dysplasia of the hip > Risk factors for developmental dysplasia of the hip: Breech Family history Oligohydramnios Packaging disorders Female sex First born child > History of ultrasonography (age, indication) > Primary method of diagnosis (clinical/ultrasonography) > Treatment history

The patients were subdivided into early (age <3 months at diagnosis, Group 1) and late (age ≥3 months, Group 2) diagnosis groups.^3^ The relative frequency of risk factors for DDH between the two groups was compared and Fisher’s exact test was performed to determine whether a significant difference was present. Group 1 was analysed further to identify the number of cases with recognised causes for the failure of initial management with a Pavlik harness such as age at onset of treatment or bilaterality.^15^

The screening practices of local maternity units were ascertained by a direct telephone questionnaire survey with members of either their medical or nursing teams.

## Results

Overall, 73 primary open reductions of the hip were performed in 55 patients over the 5-year period. After exclusions, 48 patients with 64 affected hips were eligible for the study (Table 2). Forty-one patients (85%) were female. There were no significant differences in sex distribution between the groups.

**Table 2:** Comparative analysis of the characteristics of the three groups (Group 1a: diagnosed by 6 weeks; Group 1b: diagnosed between 6 and 12 weeks; Group 2: diagnosed after 12 weeks)

		Group		
		1a (*n*=7)	1b (*n*=7)	2 (*n*=34)
Median age at diagnosis (range)		0.25 mths (0–1.5 mths)	2.25 mths (2.0–3.0 mths)	16 mths (4–60 mths)
Bilateral hip involvement		7 (100%)	2 (29%)	8 (24%)
One or more risk factors		7 (100%)	4 (57%)	4 (12%)
Neonatal hip ultrasonography		7 (100%)	3 (43%)	–
Positive clinical screening	Neonatal	4 (57%)	–	–
	6–8 weeks	–	4 (57%)	–
	8–12 months	–	–	4 (12%)

The median age at diagnosis was 14 months (range: 1 day – 5 years). There were 14 patients in Group 1 (29%) and 34 patients in Group 2 (71%). Hip pathology was detected by routine child health surveillance in 40% of the patients in this series. The other 60% presented owing to parental concerns.

The left hip was affected in 16 patients (33%), the right hip in 15 (31%) and bilateral hip involvement was found in 17 (36%). Intergroup analysis found that bilateral hip involvement was significantly more prevalent in Group 1 (71%) than in Group 2 (24%) (Fisher’s exact test, *p*=0.003).

**Figure 1:**
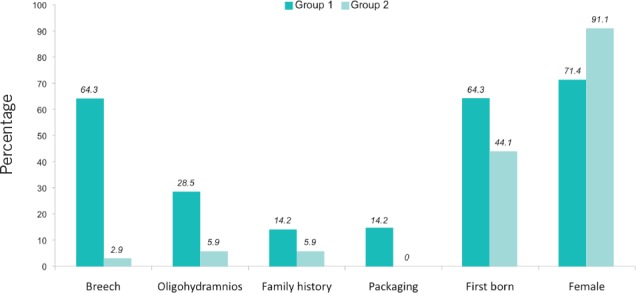
The prevalence of specific risk factors for developmental dysplasia of the hip in the two groups

The prevalence of each of the risk factors in the two groups is summarised in Figure 1. There were no significant differences in the number of first born children or girls between the two groups. These factors were included in the study as they are known risk factors for DDH^14^ but, to the best of our knowledge, they are not used as triggers for selective ultrasonography screening in the UK. The other risk factors (breech, oligohydramnios, family history and packaging disorders), which are commonly used triggers, were all significantly more prevalent in Group 1 (Fisher’s exact test, *p*<0.00001).

### Subgroup analysis

All patients in Group 1 were diagnosed by routine child health surveillance. Twelve of the fourteen children had risk factors used for selective ultrasonography. This group was subdivided based on whether Pavlik harness treatment was initiated by two months of age.

Group 1a comprised seven patients, all with bilateral hip dislocations, who received diagnosis and treatment with a Pavlik harness within six weeks. Of these, four cases were detected at the immediate postnatal clinical check and three by risk factor-based ultrasonography.

Group 1b also comprised seven patients. A common characteristic was a delay in the initiation of Pavlik harness splinting beyond nine weeks of age. Four patients did not receive their risk factor triggered ultrasonography until between 8 and 13 weeks of age, which delayed the initiation of the Pavlik harness treatment to between 9 weeks and 4 months. Three patients were detected at routine postnatal checks performed by their general practitioners between six and nine weeks of age, and subsequently referred to secondary units. Two of these three patients had never been offered ultrasonography despite having risk factors.

The median age at diagnosis of the 34 patients in Group 2 was 16 months (range: 4–60 months). Only 15% were detected during routine clinical checks by health professionals. The remainder (85%) presented as a result of parental concern, most commonly about gait abnormalities (62%) or leg length discrepancy (18%). However, multiple consultations with a general practitioner were required in 40% of these cases before the diagnosis was established. None of the patients in Group 2 received ultrasonography and only four (12%) had risk factor triggers.

The characteristics of the three groups are summarised in Table 2.

### Survey of local maternity units

The maternity units of 12 hospitals from our region were included in the survey, all of which had referred cases to our unit. All had a policy of universal neonatal clinical hip screening. The clinical examinations were performed by a senior house officer in paediatric medicine in six units, a registrar in paediatric medicine in two units and midwives in four units. All units only performed ultrasonography examination selectively: in newborns with a family history, breech presentation, oligohydramnios or abnormal clinical findings. The target time for the hip ultrasonography varied between three and five weeks of age.

## Discussion

This study demonstrates that our unit is performing open reductions of the hip predominantly for late presentation DDH rather than for failure of early non-operative methods (71% vs 29% of cases). Only 40% of patients in this series were detected by routine surveillance. These findings indicate a failure of the design and delivery of screening programmes in our region.

In this series, only bilateral cases required open reduction for failure of primary harness treatment started before six weeks. This highlights the importance of early detection and management, and, consequently, effective screening. Several authors, including Pavlik, have stressed previously the importance of commencing harness treatment early.^2,7,15^

In our study, DDH risk factors used for selective ultrasonography were significantly more prevalent in the early presentation group. This may be because ‘early’ and ‘late’ onset DDH are completely disparate entities, with differing associations, so screening for one will not detect the other.

Another explanation, which we favour, is the confounding effect of ultrasonography, which, in this series, made the early diagnosis despite a ‘normal’ clinical examination in half of the early cases. By prompting a scan, risk factors for DDH become protective against late presentation and are therefore likely to be more prevalent in those diagnosed early. This theory is supported by the finding that the two risk factors not used for selective ultrasonography screening (female sex and first born child) were the only two that were equally prevalent between the early and late groups. In our region, ultrasonography seems to be instrumental in diagnosing DDH early. Similarly, Sharpe *et al* reported that breech presentation confers protection against late presentation DDH.^16^

Even the most stringently applied selective ultrasonography screening programme would have failed to detect 88% of the late diagnosis group as they had no risk factors. To reduce late presenting DDH, an alternative screening strategy is required such as universal ultrasonography or using female sex and first born birth as selective screening triggers.

In 1989 Clarke *et al* reported that selective ultrasonography screening did not reduce the incidence of late diagnosed DDH.^17^ The same unit then demonstrated that routine universal ultrasonography screening eradicated late presenting DDH and is cost effective.^18^ Similar findings have been reported following the introduction of universal ultrasonography screening in an Austrian province (50% reduction in open reduction, 72% reduction in closed reduction).^19^ Nevertheless, the merits of universal ultrasonography screening are still questioned, with some studies suggesting selective screening provides better outcomes.^20^

Overall, 23% of cases in our series represent failures in the delivery of a selective screening process, with imaging performed late or not at all. This is a substantial proportion of patients who could possibly have avoided surgery if detected earlier.

The efficacy of selective screening programmes depends on the quality of the neonatal hip examination,^21^ which has, in turn, been shown to depend on the expertise of the examiner.^22–24^ These tests are still often performed by junior, inexperienced staff in our region and across most of the UK. Only eight cases from Group 1 (57%) were detected primarily by clinical examination and only twelve cases (25%) in the entire series. Without expert examiners, we cannot hope to emulate the success of other selective screening programmes.

In 2008 the UK National Screening Committee established the NHS Newborn and Infant Physical Examination (NIPE) programme to develop national standards for a high quality screening programme.^25^ A stated goal is a reduction in the need for surgery for DDH; an emphasis is placed on the importance of competency in clinical examination, and timeliness in investigations and referral for expert opinion.^26^ Their proposed standards are hip ultrasonography within two weeks in any newborn with positive clinical screening and within six weeks if they have a risk factor. The only compulsory risk factors for ultrasonography are a family history of a first degree relative or breech presentation.

The intentions of the programme are highly commendable and a quality framework is much needed. However, based on our study, the extent of its impact on open surgery of the hip would be a reduction of about 25%. While this would be a considerable improvement, it is a matter for debate whether this would be enough.

## Conclusions

The results of our study demonstrate that open reduction of the hip, despite neonatal hip screening, is still performed most commonly for late presentation DDH. There appears to be considerable scope for improvement in the delivery of current selective ultrasonography-based screening programmes in our region on several fronts, some of which may be addressed by NIPE.

Most late presenters do *not* have risk factors that would trigger ultrasonography in even the most stringently applied selective screening programme, making the clinical hip examination the only screening test these neonates will undergo. Consequently, it should be performed by specially trained, experienced individuals. Universal ultrasonography screening would compensate for this lack of clinical expertise and should be considered as an alternative strategy.

Inadequately designed and delivered screening is destined to fail, and children will continue to undergo open surgical reductions for DDH unnecessarily because diagnosis was too late to attempt closed methods. We feel it is our duty as orthopaedic clinicians to take the responsibility for ensuring that high standards of practice in neonatal hip screening are attained.
